# Monitoring patients’ symptom improvement in palliative care units using patient-reported outcomes: a multicenter prospective observational study

**DOI:** 10.1186/s12904-026-01990-9

**Published:** 2026-01-22

**Authors:** Natsuko Katsube, Akihiro Sakashita, Kyoko Kamohara, Kousei Adachi, Ritsuko Yabuki, Akira Inoue, Mamiko Sato, Syun Koike, Hirofumi Abo, Kento Masukawa, Yasuo Shima, Mitsunori Miyashita

**Affiliations:** 1 Department of Palliative Nursing, Health Sciences, Graduate School of Medicine, Tohoku University, 2-1 Seiryo-Machi, Aoba-Ku, Sendai, 980-8575 Japan; 2 Division of Palliative Medicine, Department of Internal Medicine, Hyogo Prefectural Harima-Himeji General Medical Center, Himeji, Japan; 3 Tsujinaka Hospital Kashiwanoha, Chiba, Japan; 4 Kobe Adventist Hospital, Kobe, Japan; 5 Adventist Medical Center, Okinawa, Japan; 6 Department of Palliative Medicine, Tsukuba Medical Center Hospital, Tsukuba, Japan; 7 Department of Palliative Medicine, Tohoku University Hospital, Sendai, Japan; 8 Public Tomioka General Hospital, Gunma, Japan; 9 Department of Palliative Medicine, Rokko Hospital, Kobe, Japan

**Keywords:** Palliative care, Palliative care unit, Patient reported outcome, Symptom management, Quality of health care

## Abstract

**Background:**

The use of patient-reported outcomes (PROs) can facilitate the reduction of the severity of patient symptoms. Several countries have implemented projects that routinely use PROs in palliative care settings, resulting in increased patient symptom improvement rates. In Japan, a pilot study of hospital-based palliative care teams was conducted in 2021; however, no study has been conducted in palliative care units (PCUs). This study assessed patient symptom improvement rates using PROs and evaluated the feasibility of routine PRO assessment and data collection in PCUs in Japan.

**Methods:**

We conducted a multicenter, prospective, observational study in eight PCUs. Patients newly admitted to PCUs between June and September 2024 were included in this study. Based on the analysis requirement of 369 PRO responders and an assumed 60% response rate, 615 participants were targeted for enrollment (369/0.60). Data on four symptoms (pain, shortness of breath, nausea, and worries or concerns) were collected weekly using the Integrated Palliative Outcome Scale (IPOS) from admission to week 4.

**Results:**

A total of 550 patients were admitted to the PCUs; 388 self-reported patients were included in the analysis. The PRO response rate was > 70% at all timepoints. The IPOS score decreased statistically only between admission and week 1 (pain, *p* < 0.001; shortness of breath, *p* < 0.001; nausea, *p* = 0.001; worries or concerns, *p* < 0.001). A 1-point decrease in IPOS scores was observed for pain (from 2 to 1), shortness of breath (from 2 to 1), worries or concerns (from 2 to 1) from admission to week 1, and shortness of breath from week 3 to week 4 (from 1 to 0). In the “severe/moderate to absent/mild” and “keep absent/mild” categories, benchmark improvement rates were achieved only for nausea (70.7%; 90.0%).

**Conclusions:**

A 1-week regular evaluation using PROs may be feasible in PCUs in Japan. The greatest improvement in symptom scores occurred within the first week following PCU admission, with an observed plateau in subsequent weeks, suggesting that the first week may be important for assessing the quality of care in PCUs.

**Supplementary Information:**

The online version contains supplementary material available at 10.1186/s12904-026-01990-9.

## Background

Patient-reported outcomes (PROs) are reports on a patient’s health status that come directly from the patient, without interpretation by a clinician or anyone else [1]. Many patients with terminal cancer experience multiple symptoms [2–4], and medical professionals often underestimate the severity of these symptoms [5, 6]. Therefore, the use of PROs is recommended for identifying palliative care needs in both clinical practice and research [7]. PROs help identify and manage symptoms, ultimately reducing their severity [8–10]. 

Some countries have implemented projects that routinely use PROs in palliative care settings. The Palliative Care Outcome Collaboration (PCOC) in Australia monitors patient symptom improvement rates in palliative care services using PROs, which has led to improved rates nationwide [11, 12]. In the PCOC, patients’ symptoms are evaluated daily using PROs and submitted to the project office every 6 months for analysis. Each palliative care service uses the analysis results to implement measures to improve patient care. Similar projects have been initiated in Asia, including Taiwan [13]. 

In Japan, efforts to assess and improve the quality of specialized palliative care have not focused on patient outcomes, such as actual symptom alleviation. Therefore, a project was initiated to establish a system for evaluating the quality of specialized palliative care using PROs. A 2021 pilot study involving eight palliative care teams demonstrated the effectiveness of hospital-based palliative care teams in improving patient symptoms and highlighted challenges in PRO utilization [14, 15]. However, no investigations have been conducted in Japanese inpatient palliative care units (PCUs), which are instrumental for patients with terminal cancer. One survey reported that only 11% of PCUs routinely used PROs [16]. The introduction of PROs may place additional burden on medical professionals and patients [17, 18]. Previous studies have revealed that factors such as defined aims, processes, and responsibilities, as well as and the need for training, facilitators, and digital implementation, are crucial [19–21]. However, cultural differences in attitudes toward research and palliative care may influence the willingness of both patients and medical professionals to use PROs, making it necessary to conduct surveys in Japanese inpatient PCUs [22]. 

Therefore, this study aimed to clarify patient symptom improvement rates on using PROs and to evaluate the feasibility of regular PRO assessment and data collection in PCUs in Japan.

## Methods

This multicenter, prospective, observational study was reported in accordance with the STROBE guidelines [23]. Data were collected from eight PCU facilities in Japan, selected through convenience sampling from member facilities of the *Hospice Palliative Care Japan* association. One facility had 48 beds, while the others were smaller units with capacities ranging from 18 to 24 beds. Only one facility had routinely used PROs before the initiation of this study.

Before the study, a manual detailing objectives, procedures, assessment scales, scheduling, and data management was distributed to facility principal investigators. A subsequent briefing session provided verbal explanations and addressed questions. Principal investigators were tasked with internally sharing this information with their staff. Continuous communication was maintained via email for any subsequent inquiries.

### Participants

We enrolled all inpatients admitted to the PCUs between June 1 and September 30, 2024. The study also included patients transferred within the same hospital’s PCU and those readmitted during the survey period. Because the purpose of this study was to investigate symptom improvement following admission to a PCU, we did not collect information on whether an admission was a first admission or a readmission.

### Sample size calculation

To calculate the sample size, we assumed that 60% of inpatients had moderate or greater levels of pain [2], revealing that 369 participants were required to achieve a 95% confidence interval with a standard error of 0.05. Furthermore, assuming a PRO response rate of 60% with reference to the PCOC data [24], the sample size was finalized as 615 (369 / 0.60). Based on the number of monthly hospitalizations at each site, we determined that the required number of cases could be reached within 4 months; therefore, the study duration was set at 4 months.

### Measurements

We examined symptom improvement rates using PROs recorded 1 week after PCU admission, as well as changes in IPOS scores from admission to 4 weeks post-admission. We assessed the feasibility of regular PRO evaluation and data collection using valid response and PRO response rates.

#### PROs

PROs were evaluated using the Japanese version of the Integrated Palliative Care Outcome Scale (IPOS) [25]. Although we provided the option to use Edmonton Symptom Assessment System (ESAS) instead of IPOS, depending on each facility’s ease of use, ESAS was not utilized [26]. IPOS is a palliative care symptom assessment scale that can be used by both patients and healthcare professionals, with verified reliability and validity in their Japanese version [27, 28]. Each item of the IPOS is evaluated on a 5-point scale ranging from 0 to 4, with higher scores indicating more severe symptoms. The IPOS evaluates symptoms over the past 3 days. Each assessment was performed 7 days (± 2 days) after the previous one. We decided to assess only four symptoms: “pain,” “shortness of breath,” and “nausea” as physical symptoms, which are common among patients with cancer [29], and “worries or concerns” as a psychosocial problem. “Pain”, “shortness of breath”, and “nausea” were assessed using items from the IPOS and ESAS, and, following discussion among the researchers, “worries or concerns” was added as an additional item [15]. Because we wanted to reduce the burden on both patients and medical professionals and because this was a pilot study, the questionnaire items were consolidated into four domains. Consequently, psychological and social aspects were combined into a single item “worries or concerns.” If patients were unable to evaluate their symptoms themselves, the reasons were recorded by medical professionals. The questionnaire was developed specifically for this study (Supplementary Material 1).

In this study, if “patients verbally described their symptoms, and the medical professionals filled out the questionnaire,” the inputs were also treated as PROs, as we intended to incorporate the patient’s suffering as much as possible into the assessment. Additionally, patients in palliative care settings may have difficulty completing questionnaires without support. If patients had difficulty answering independently, assistance from family members, caregivers, or medical professionals was permitted; however, proxy responses by family members or caregivers were not allowed. If the patient could not report any symptoms, a medical professional assessed the patient’s symptoms. Patients were considered capable of self-assessment when they were able to write down or verbally express the severity of their own symptoms. Proxy assessment by medical professionals was deemed unfeasible when no observable responses were provided by the patient (e.g., unresponsive or comatose).

#### Patient characteristics

We obtained the patients’ Eastern Cooperative Oncology Group performance status as an indicator of their overall health. Additionally, Palliative Care Phase was obtained to separately assess the timeliness of care [30, 31]. We collected data on admission date, sex, age, disease (mainly life-limiting disease), cancer site, recurrence or metastasis, and purpose of admission as demographic and clinical information at PCU admission. We also collected discharge dates and outcomes at discharge.

#### Patient symptom improvement rates

We used only PRO data for these calculations. We calculated the patient symptom improvement rates 1 week after admission using the following three categories:

1) Severe to moderate or less: the proportion of patients whose symptoms improved to moderate or less after 1 week among those with severe symptoms at admission;

2) Severe/moderate to absent/mild: the proportion of patients whose symptoms improved and were absent or mild after 1 week among those with severe or moderate symptoms at admission; and.

3) Keep absent/mild: the proportion of patients whose symptoms remained absent or mild after 1 week among those with absent or mild symptoms at admission.

We focused on the improvement rates 1 week after admission to investigate the effects of prompt care provided after admission. Benchmarks were set at ≥ 60% for transitions from “severe/moderate to absent/mild,” and ≥ 90% for symptoms that “keep absent/mild,” based on PCOC standards [11]. We did not set a benchmark for “severe to moderate or less” due to a lack of previous research conducted in PCUs for this category. Symptom severity levels were defined as severe (IPOS: 3–4), moderate (IPOS: 2), or mild (IPOS: 1) [25]. 

#### Feasibility of regular PRO evaluation and data collection

Patients who could not undergo symptom assessment by medical professionals were excluded from the valid response rate calculation. The PRO response rate was defined as the proportion of patients who responded themselves among those who provided valid responses. We set a target of ≥ 90% for the valid response rate, as missing data ≥ 10% is likely to introduce bias [32]. The target PRO response rate was set at 60% based on previous studies [24]. 

### Data collection

We collected data on the IPOS, performance status, and Palliative Care Phase at admission and every week until week 4. New patients were enrolled until the end of September, and the follow-up period continuted until October 7, after which data collection was completed.

Data from the completed questionnaires were transcribed into an Excel-based format prepared by the study coordinators. Facilities were also allowed to develop their own electronic medical record system and directly input patients’ symptoms according to their preferences. All data were submitted to the study office.

### Statistical analysis

Regarding the changes in IPOS scores, the median (interquartile range) IPOS scores at each timepoint was calculated. Differences between admission and week 1, week 1 and week 2, week 2 and week 3, and week 3 and week 4 were tested using the Bonferroni-corrected Wilcoxon signed-rank test. Statistical significance was set at *p* < 0.0125. Statistical analyses were performed using SAS 9.4 (SAS Institute Inc., Cary, NC, USA). As only a few studies have reported minimal clinically important differences for some IPOS items and no consensus has been reached [33], clinical significance was not considered.

To identify predictors of non-completion of PROs, we initially planned a multinomial logistic regression analysis across three categories: patients with completed PROs, patients with unassessed PROs but with clinician assessment, and patients with unassessed PROs without any assessment. However, owing to the limited number of patients in the non-evaluated group (*n* = 27), we opted for a binomial logistic regression analysis. In this revised model, we compared the clinician-assessed group against the PRO-completed group (reference), excluding the non-evaluated group from the analysis. A backward elimination method (removal criterion: *p* > 0.20) was applied to determine the best-fit binomial logistic regression model.

### Ethical considerations

The Ethics Review Committee of the Tohoku University Graduate School of Medicine (No. 2023-1-826) approved this study. The study qualified as a noninvasive observational study according to current Japanese law (data collected during routine practice). The requirement for informed consent from the patients was waived in accordance with the Japanese guidelines for noninvasive observational studies; however, notifications about the study were published, and participants were ensured the opportunity to withdraw from participation.

## Results

### Patient characteristics

**Fig. 1:**
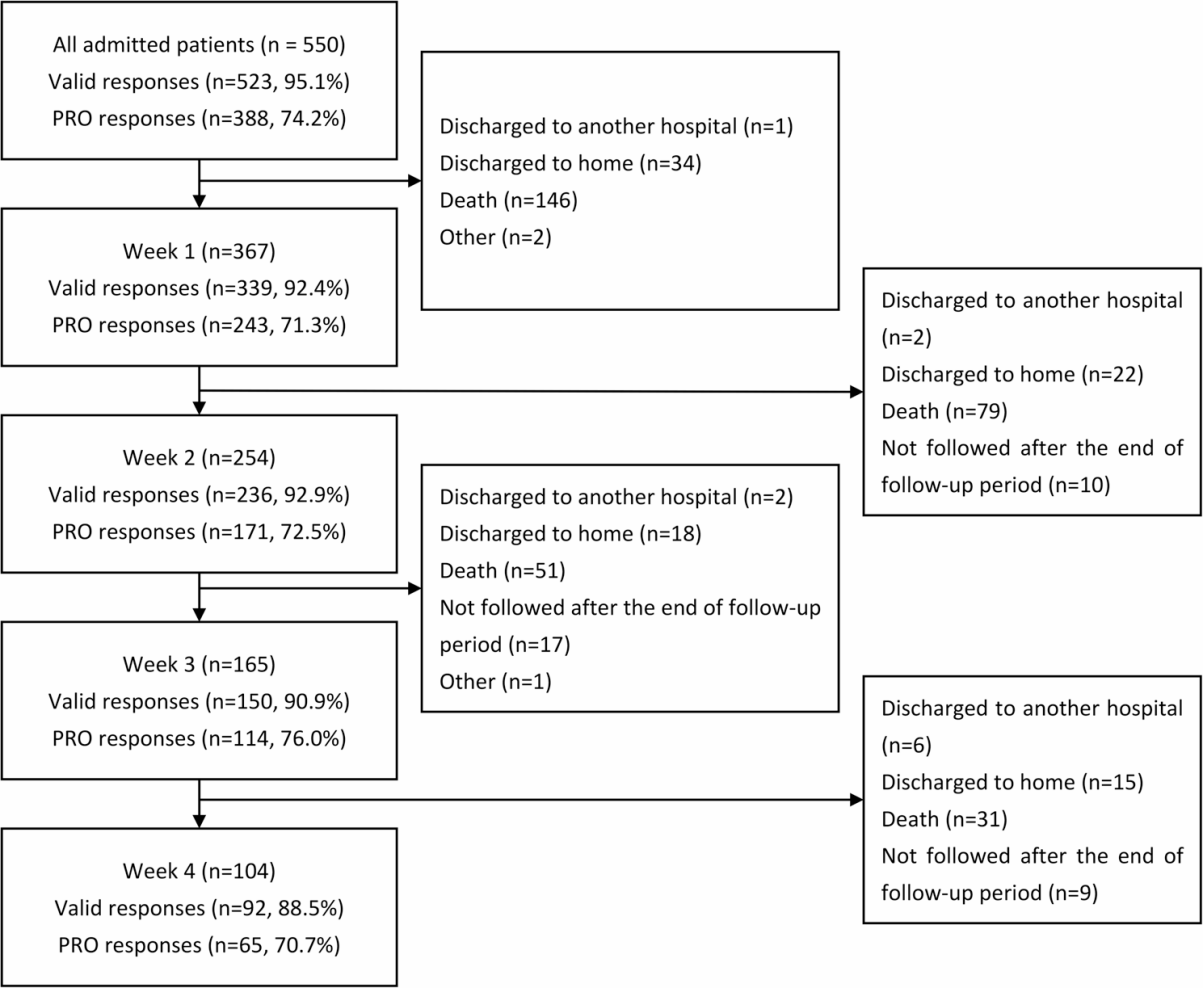
Patient flow diagram and PRO response rates. Valid response rate: The proportion of patients with symptom evaluations conducted by medical professionals or patients. Patients with no evaluable data were excluded. PRO response rate: The proportion of patients who responded by themselves among those who provided valid responses. PRO, patient-reported outcome

Figure 1 shows a patient flow diagram. Overall, a total of 550 patients were admitted to the PCU during the study period. The analysis included 388 patients who responded by themselves at admission. Table 1 lists the characteristics of all patients and those with completed PROs. The mean age ± standard deviation was 73.9 ± 11.7 years, and 56.2% of patients were male. Notably, 98.2% of patients had cancer, and 62.1% of patients died, which was the most common outcome among patients with completed PROs. The average hospital stay among these patients was 17.7 ± 15.3 days.

**Table 1:** Patient characteristics

		All patients (*n* = 550)		Patients with completed PROs (*n* = 388)	
		*n*	%	*n*	%
Facility	1	69	12.6	44	11.3
	2	64	11.6	39	10.1
	3	139	25.3	106	27.3
	4	33	6.0	29	7.5
	5	36	6.6	19	4.9
	6	43	7.8	20	5.2
	7	59	10.7	38	9.8
	8	107	19.5	93	24.0
Sex	Male	303	55.1	218	56.2
	Female	247	44.9	170	43.8
Age	Mean (± SD)	74.5	11.3	73.9	11.7
Disease	Cancer	536	97.5	381	98.2
	Other	14	2.5	7	1.8
Cancer site	Lung	94	17.5	62	16.3
	Stomach	41	7.7	32	8.4
	Esophageal	19	3.5	14	3.7
	Liver/biliary tract	39	7.3	29	7.6
	Pancreas	63	11.8	48	12.6
	Breast	28	5.2	20	5.3
	Urinary tract	53	9.9	36	9.5
	Head and neck	26	4.9	18	4.7
	Uterus/ovary	34	6.3	24	6.3
	Hematopoietic/lymphoid tissues	15	2.8	12	3.2
	Soft tissue	5	0.9	4	1.1
	Skin	3	0.6	2	0.5
	Brain	7	1.3	4	1.1
	Colon	87	16.2	63	16.5
	Other	22	4.1	13	3.4
Recurrence or metastasis (cancer only)		485	90.8	352	90.7
Purpose of admission	Symptom management	504	91.6	368	94.9
	End of life care	224	40.7	138	35.6
	Respite	17	3.1	13	3.4
	Other	14	2.6	9	2.3
Palliative Care Phase	Stable	29	5.4	38	13.9
	Unstable	266	49.4	95	34.7
	Deteriorating	190	35.3	119	43.4
	Dying	53	9.9	22	8.0
Performance status	0	2	0.4	1	0.4
	1	17	3.2	9	3.3
	2	46	8.5	34	12.4
	3	232	43.0	132	48.2
	4	243	45.0	98	35.8
Outcome	Discharged to another hospital	20	3.6	16	4.1
	Discharged home	105	19.1	84	21.7
	Death	361	65.6	241	62.1
	Other	64	11.6	47	12.1
Length of hospital stay	Mean (SD)	16.3	14.9	17.7	15.3
	0 day	13	2.4	4	1.0
	1–7 days	183	33.3	114	29.4
	8–14 days	118	21.5	93	24.0
	15–21 days	86	15.6	60	15.5
	22–28 days	58	10.6	43	11.1
	29+ days	92	16.7	74	19.1

*SD* Standard deviation

**Fig. 2:**
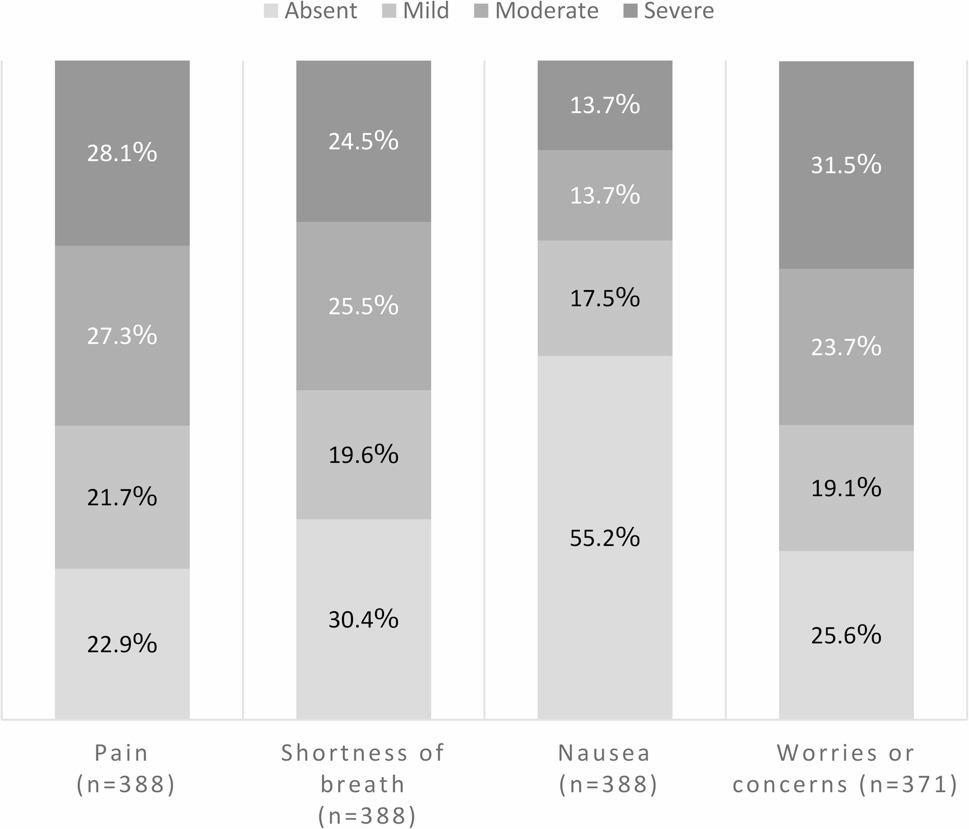
Prevalence and severity of symptoms at admission among patients with completed PROs. Distribution of symptom severity at PCU admission (n = 388). Absent: IPOS = 0; Mild: IPOS = 1; Moderate: IPOS = 2; Severe: 3≤ IPOS ≤4. IPOS, Integrated Palliative Care Outcome Scale; PCU, palliative care unit

Figure 2 displays the prevalence and severity of symptoms at admission among patients with completed PROs. The results for all patients are presented in Additional file 1. The prevalence rates were as follows: pain, 77.1% (*n* = 299/388); shortness of breath, 69.6% (*n* = 270/388); nausea, 44.8% (*n* = 174/388); and worries or concerns, 74.4% (*n* = 276/371). The proportion of patients with moderate or severe symptoms was as follows: pain, 55.4% (*n* = 215/388); shortness of breath, 50.0% (*n* = 194/388); nausea, 27.3% (*n* = 106/388); and worries or concerns, 55.3% (*n* = 205/371).

### Patient symptom improvement rates using PROs

**Fig. 3:**
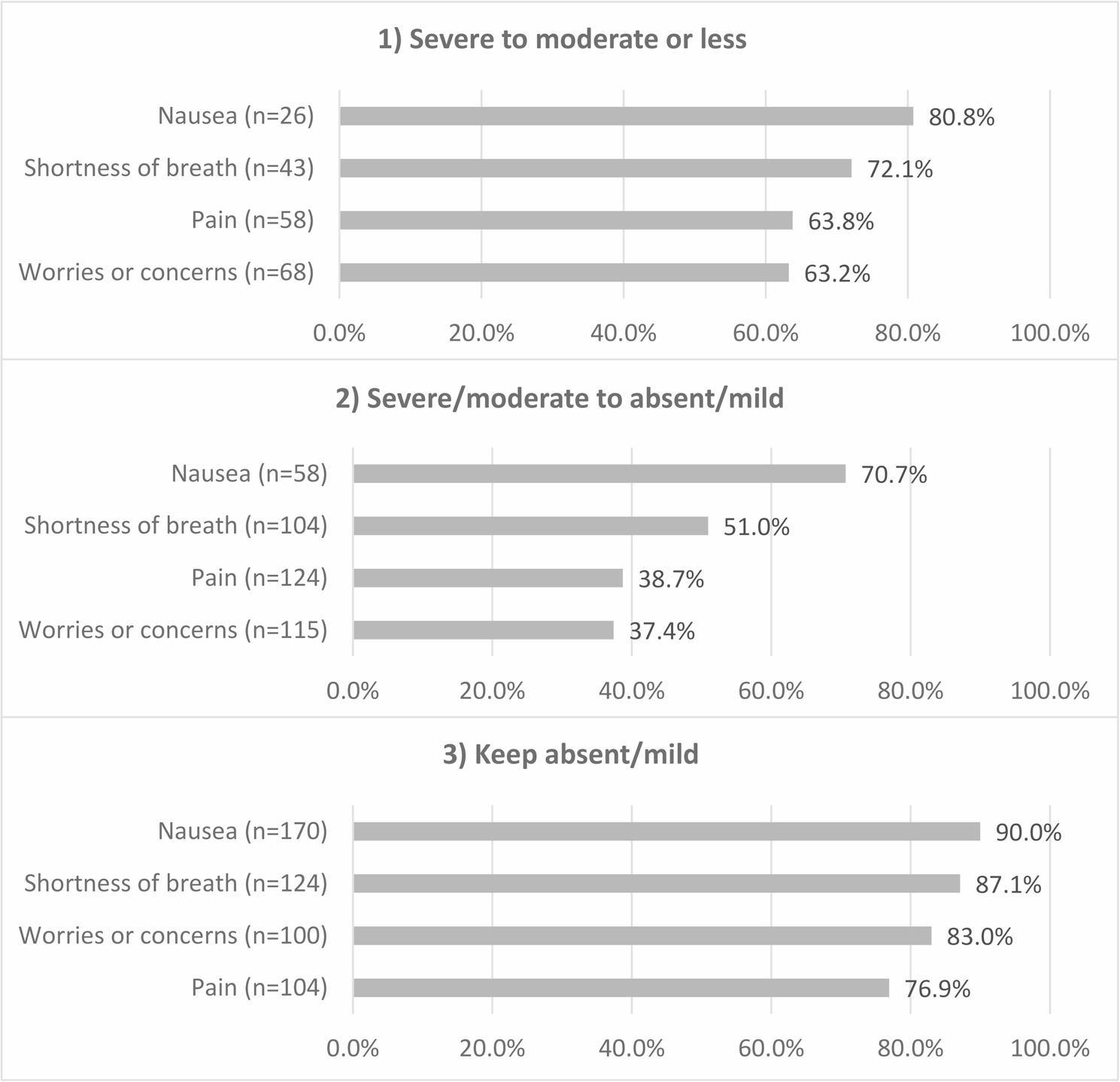
Symptom improvement rates 1 week after PCU admission among patients with completed PROs.We focused on the improvement rates 1 week after admission to investigate the effects of prompt care on patient symptoms:1) Severe to moderate or less: the proportion of patients whose symptoms improved to moderate or less after 1 week among those with severe symptoms at admission;2) Severe/moderate to absent/mild: the proportion of patients whose symptoms improved and were absent or mild after 1 week among those with severe or moderate symptoms at admission; and3) Keep absent/mild: the proportion of patients whose symptoms remained absent or mild after 1 week among those with absent or mild symptoms at admission.n: number of patients with severe, severe/moderate, or mild symptoms at admission. PRO, patient-reported outcome; PCU, palliative care unit

Figure 3 shows the patient symptom improvement rates for each symptom 1 week after admission among patients with completed PROs. The results for all patients are presented in Additional file 2. The rates for “severe to moderate or less” were as follows: nausea, 80.8% (*n* = 21/26); shortness of breath, 72.1% (*n* = 31/43); pain, 63.8% (*n* = 37/58); and worries or concerns, 63.2% (*n* = 43/68). In the “severe/moderate to absent/mild” and “keep absent/mild” categories, benchmark improvement rates were achieved only for nausea (70.7%, *n* = 41/58; 90.0%, *n* = 153/170).

**Fig. 4:**
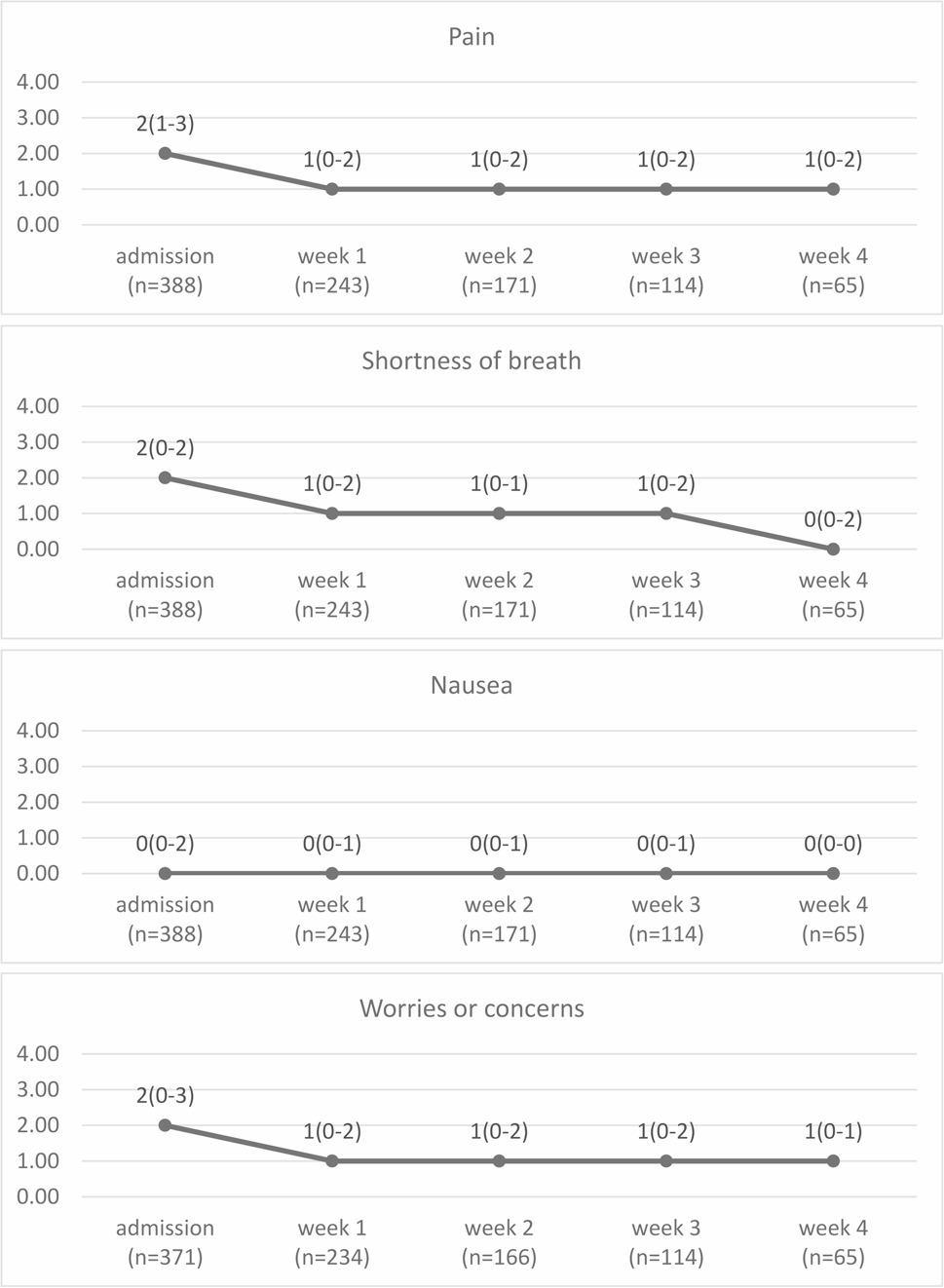
Changes in IPOS scores from admission to week 4 among patients with completed PROs. The values at each timepoint in the figure represent the median with the interquartile range (IQR). IPOS, Integrated Palliative Care Outcome Scale; PRO, patient-reported outcome

Figure 4 demonstrates the change in the IPOS score from admission to week 4 among patients with completed PROs. The results for all patients are presented in Additional file 3. Statistically significant differences from admission to week 1 were observed in pain (S = -1562, *p* < 0.001), shortness of breath (S = -1468.5, *p* < 0.001), nausea (S = -817, *p* = 0.001), and worries or concerns (S = -1736.5, *p* < 0.001). No statistically significant changes were observed in any symptom at the other timepoints. A 1-point decrease was observed for pain (from 2 to 1), shortness of breath (from 2 to 1), worries or concerns from admission (from 2 to 1) to week 1, and shortness of breath from week 3 to week 4 (from 1 to 0).

### Feasibility of regular PRO evaluation and data collection

The target valid response rate of 90% was achieved through week 3 and was maintained by week 4 (Fig. 1). The PRO response rate exceeded the target of 60% at all timepoints. The most common reasons patients could not answer questions by themselves were impaired consciousness (cognitive impairment), physical distress, and refusal (Table 2). Table 3 shows the baseline characteristics of patients who were unable to complete the PRO assessments. Table 4 summarizes the results of the binomial logistic regression analysis for factors predicting clinician-based evaluation. The backward elimination procedure initially identified Facility, Age, Recurrence or metastasis, Purpose of admission, Palliative Care Phase, Performance status, Outcome, and Length of hospital stay as candidate variables. The final model identified the following as independent predictors: Facilities 3, 4, and 8 (all *p* ≤ 0.01); Recurrence or metastasis (AOR = 0.27; 95% CI: 0.11–0.66; *p* = 0.04); Palliative Care Phase (Dying) (AOR = 8.53; 95% CI: 1.85–39.35; *p* = 0.01); Performance status 4 (AOR = 15.32; 95% CI: 1.63–143.74; *p* = 0.02); and Length of hospital stay (AOR = 0.98; 95% CI: 0.96–1.00; *p* = 0.02).

**Table 2:** Reasons for unassessed PROs and for neither PRO nor clinician assessment (n)

	Unassessed PROs (*n*)					Neither PRO nor clinician assessed (*n*)					
	Admission	Week 1	Week 2	Week 3	Week 4	Admission	Week 1	Week 2	Week 3	Week 4	
Total	135	96	65	36	27	27	28	18	15	12	
Refusal	0	3	0	0	2	1	0	0	0	0	
Impaired consciousness (cognitive impairment)	100	80	56	30	19	23	24	15	15	12	
Sedation	0	1	1	2	1	0	0	0	0	0	
Young child	0	0	0	0	0	0	0	0	0	0	
Physical distress	31	9	3	4	3	0	1	1	0	0	
Psychological distress	0	1	0	0	0	0	0	0	0	0	
Other	4	2	5	0	2	3	3	2	0	0	

The values for “death” and “discharge” were zero because the study only included patients who were alive or hospitalized at the time of evaluation

Other reasons for unassessed PROs included near-death condition and missed patient interviews

Other reasons for the lack of both PRO and clinician assessments included near-death condition, failure to document, and overnight leave

**Table 3:** Baseline characteristics of patients who were unable to complete the PRO assessments

		(a) Unassessed PROs without any assessment (*n* = 27)		(b) Unassessed PROs with clinician assessment (*n* = 135)	
		*n*	%	*n*	%
Facility	1	0	0	25	18.5
	2	4	14.8	21	15.6
	3	0	0	33	24.4
	4	3	11.1	1	0.7
	5	8	29.6	9	6.7
	6	12	44.4	11	8.2
	7	0	0	21	15.6
	8	0	0	14	10.4
Sex	Male	14	51.9	71	52.6
	Female	13	48.2	64	47.4
Age	Mean (± SD)	78.2	11.1	75.5	10.2
Disease	Cancer	27	100	128	94.8
	Other	0	0	7	5.2
Cancer site	Lung	7	25.9	25	19.5
	Stomach	2	7.4	7	5.5
	Esophageal	0	0	5	3.9
	Liver/biliary tract	2	7.4	8	6.3
	Pancreas	3	11.1	12	9.4
	Breast	1	3.7	7	5.5
	Urinary tract	2	7.4	15	11.7
	Head and neck	3	11.1	5	3.9
	Uterus/ovary	3	11.1	7	5.5
	Hematopoietic/lymphoid tissues	0	0	3	2.3
	Soft tissue	0	0	1	0.8
	Skin	0	0	1	0.8
	Brain	1	3.7	2	1.6
	Colon	3	11.1	21	16.4
	Other	0	0	9	7.0
Recurrence or metastasis (cancer only)		20	74.1	113	88.3
Purpose of admission	Symptom management	22	81.5	114	84.4
	End of life care	9	33.3	77	57.0
	Respite	0	0	4	3.0
	Other	0	0	5	3.7
Palliative Care Phase	Stable	0	0	4	3.0
	Unstable	4	26.7	54	40
	Deteriorating	5	33.3	40	29.6
	Dying	6	40	37	27.4
Performance status	0	0	0	0	0
	1	0	0	1	0.7
	2	0	0	5	3.7
	3	3	17.7	36	26.7
	4	14	82.4	93	68.9
Outcome	Discharged to another hospital	3	11.1	1	0.7
	Discharged home	0	0	21	15.6
	Death	21	77.8	99	73.3
	Other	3	11.1	14	10.4
Length of hospital stay	Mean (SD)	20.8	18.7	11.5	11.8

When PROs were not assessable, clinician assessments were performed instead

*SD* Standard deviation

**Table 4:** Binomial logistic regression analysis for factors predicting clinician-based evaluation

Effect		Reference	Adjusted OR	95% Cl		*p*
Facility	2	1	0.99	0.40	2.49	0.986
	3	1	0.14	0.06	0.34	< 0.001
	4	1	0.06	0.01	0.55	0.013
	5	1	0.28	0.08	1.04	0.057
	6	1	1.62	0.56	4.69	0.371
	7	1	0.57	0.22	1.48	0.247
	8	1	0.10	0.04	0.25	< 0.001
Age			1.02	1.00	1.04	0.108
Recurrence or metastasis (cancer only)	Yes	No	0.27	0.11	0.66	0.004
Purpose of admission (Symptom management)	Yes	No	2.01	0.72	5.62	0.182
Purpose of admission (End of life care)	Yes	No	0.59	0.29	1.18	0.134
Purpose of admission (Other)	Yes	No	0.39	0.09	1.61	0.192
Palliative Care Phase	Unstable	Stable	1.30	0.36	4.74	0.691
	Deteriorating	Stable	0.92	0.25	3.43	0.904
	Dying	Stable	8.53	1.85	39.35	0.006
Performance status	2	0, 1	3.05	0.29	31.97	0.353
	3	0, 1	4.00	0.44	36.14	0.217
	4	0, 1	15.32	1.63	143.74	0.017
Length of hospital stay			0.98	0.96	1.00	0.024

Predictors of non-completion of PROs were identified using binomial logistic regression with backward elimination (removal criterion: *P* > 0.20). Due to the small sample size (*n* = 27), the non-evaluated group was excluded, and the clinician-assessed group was compared against the PRO-completed group (reference)

The PS 0 and PS 1 categories were merged to ensure statistical stability during the analysis, because only two patients had a PS of 0

Model performance was assessed using the Hosmer-Lemeshow test (*p* = 0.1913) and the C-statistic (*p* = 0.842)

*OR* Odds ratio, *CI* Confidence interval

## Discussion

To the best of our knowledge, this is the first prospective study to investigate symptom changes in PCU patients at multiple facilities in Japan. The main findings of this study are as follows: (1) regular PRO evaluations and data collection are feasible to perform in Japanese PCUs, (2) the first week after admission may be important for evaluating the effect on symptom alleviation in the PCU, and (3) benchmark for symptom improvement was met only for nausea 1 week after admission.

This study achieved PRO response rates similar to those reported in previous studies [15, 24], suggesting that regular PRO evaluations and data collection are feasible in Japanese PCUs. This may be attributable to the study’s focus on only four survey items. A previous study in Japan revealed that using all items in the IPOS or ESAS places a burden on medical professionals [15]. Other reports have also indicated that using several PRO-related items places a burden on both patients and medical professionals [17, 18]. Therefore, we plan to begin with a minimal set of items and gradually expand the scope in future studies. In this study, the primary reasons for patients being unable to complete PROs were impaired consciousness (cognitive impairment) and physical distress. Additionally, compared with patients with completed PROs, patients with unassessed PROs included a higher proportion with recurrence or metastasis, “Dying” as the Palliative Care Phase, performance status 4, and a shorter length of hospital stay. This may indicate that patients with unassessed PROs were more likely to be close to end-of-life care. Previous studies indicate that illness, cognitive impairment, and deterioration during the dying phase make PROs challenging to use in palliative care [7, 34, 35]. Furthermore, as PRO response rates varied across facilities, we plan to investigate differences based on facility characteristics in future studies.

One week after admission may be an important time to evaluate the effects on symptom alleviation in PCUs. In this study, statistical improvements were observed between admission and week 1. From week 2 onward, the trend became nearly flat. A previous study found that five items, including pain and shortness of breath, improved statistically and clinically 1 week following the start of intervention by the palliative care team [14]. A multi-institutional study on patients with cancer receiving specialized palliative care found that nearly all patients were satisfied with their pain management within 1 week [36]. Therefore, 1 week may be sufficient to evaluate the effectiveness of specialized palliative care. Symptom changes 1 week after admission is a valid indicator for objectively evaluating PCU effectiveness, although evaluating symptoms using PROs throughout the hospital stay remains desirable.

Regarding patient symptom improvement rates, a significant improvement was observed only for nausea. Our results were similar to those of previous studies conducted in Japan and Australia [11, 14]. However, the improvement rate in shortness of breath classified as “severe to moderate or less” was 10% lower than that reported in a previous Japanese study [14]. This is because the present study included more patients with terminal cancer [7]. The pain improvement rate in the “severe/moderate to absent/mild” and “keep absent/mild” categories were approximately 20% lower than that reported in Australia [11]. This study did not investigate interventions for these symptoms; therefore, further research is needed to determine the cause of this difference. We plan to work on enhancing patient symptom improvement rates in the future.

This study had several limitations. First, our results could not be generalized to all PCUs in Japan due to the small number of participating facilities. Second, as that the sample size calculation required 369 PRO responses, the target was not achieved between weeks 1 and 4. Consequently, the number of patients may have been insufficient to demonstrate significant symptom improvement during this period. Third, the item “worries or concerns” used in this study has not been validated and should be interpreted with caution, as it may encompass multiple types of psychosocial distress. Fourth, although PROs are defined as any reports on the status of a patient’s health condition that come directly from the patient without interpretation [1], this study also treated inputs recorded when “patients verbally described their symptoms and medical professionals filled out the questionnaire” as PROs. While this definition was communicated to all evaluators, no formal training was provided. Consequently, the evaluation methods may have differed between facilities. Therefore, the implementation of standardized training will be considered in future studies. Nonetheless, the collected data can be used for before-and-after comparisons and improvement measures at each facility. Standardizing evaluation methods within each facility would facilitate the effective implementation of this project. Fifth, for the change in IPOS scores from admission to week 4 among patients with completed PROs (Fig. 4), the death of patients with severe symptoms may have influenced symptom improvement at 1 week (Additional file 4). Finally, as this study lacked detailed patient data, such as information on opioid use, at the initial benchmarking, it was not possible to investigate the causes of symptom improvement or to perform inter-facility comparisons. In the future, the data obtained by this study are intended to be used by each facility for their own symptom improvement activities. Therefore, detailed patient data were intentionally not collected in this pilot study.

## Conclusions

The PRO response rates were > 70% at all timepoints, and a 1-week regular evaluation using PROs may be feasible in PCUs in Japan. The greatest improvement in symptom scores occurred within the first week following PCU admission, with an observed plateau in subsequent weeks, suggesting that the first week may be important for assessing the quality of care in PCUs. 

## Supplementary Information

Supplementary Material 1: Questionnaire for this study

Additional file 1: Prevalence and severity of symptoms at admission among all patients.

Additional file 2: Symptom improvement rates 1 week after Admission among all patients.

Additional file 3: Changes in IPOS scores from admission to week 4 among all patients.

Additional file 4: IPOS scores in patients who completed PRO assessments and died during the following week

## Data Availability

The datasets used and analyzed during the current study are available from the corresponding author on reasonable request.

## Abbreviations

PROs: Patient-Reported Outcomes
PCUs: Palliative Care Units
PCOC: Palliative Care Outcomes Collaboration
IPOS: Integrated Palliative Care Outcome Scale
ESAS: Edmonton Symptom Assessment System
SD: Standard deviation
AOR: Adjusted odds ratio
IQR: Interquartile range
95% Cl: 95% confidence interval
