# Supplementary material for: Monitoring patients’ symptom improvement in palliative care units using patient-reported outcomes: a multicenter prospective observational study
Source: BMC Palliat Care. 2026 Jan 22;25:44. doi: 10.1186/s12904-026-01990-9 (PMC12910949; doi:10.1186/s12904-026-01990-9)
Supplement: Supplementary file 1 — Supplementary Material 1: Questionnaire for this study [file 12904_2026_1990_MOESM1_ESM.pdf]

Supplementary Material 1. Questionnaire for this study

※Use this space for facility notes:

**ID**		**patient name**	
**sex**	1.male 2.female	**age**	
**admission date**	/ /		

**disease** (mainly life-limiting disease)	**Type of primary diagnosis**	1.Cancer 2.AIDS 3.Other（ ）			
	**cancer site**(Select one of the following:)				
	1.Lung	2.Stomach	3.Esophageal	4.Liver/Biliary Tract	5.Pancreas
	6.Breast	7.Urinary tract	8.Head and neck	9.Uterus/ovary	10.Hematopoietic/lymphoid tissues
	11.Soft tissue	12.Skin	13.Brain	14.Colon	15.Other( )
	**recurrence or metastasis** (cancer only)	□Yes □No			
**purpose of admission**（Select all that apply:）	1.Symptom management 2.End of life care 3.Respite 4.Other（ ）				

	Week0	Week1	Week2	Week3	Week4
**Date of assessment**（MM/DD）					
**Symptom assessor** (Please mark one option:)	Clinician ・ Patient	Clinician ・ Patient	Clinician ・ Patient	Clinician ・ Patient	Clinician ・ Patient
**A reason a patient could not answer questions by themselves**（Select one of the following:）<br>1.refusal 2.impaired consciousness (cognitive impairment) 3.Sedation 4.Young child 5.physical distress 6.Psychological distress 7.Death 8.Discharge 9.Other					
**If 9. Other, please specify:**					
**Symptom assessment** IPOS（0－4、Not applicable×） or ESAS（0－10、Not applicable×）					
**pain**					
**shortness of breath**					
**nausea**					
**worries or concerns**					
**other 1**：（ ）					
**other 2**：（ ）					
**other 3**：（ ）					
**Palliative care phase**(1-4,Not applicable×)					
**Performance status**(0-4,Not applicable×)					

**Date of discharge**	/ /
**Outcome**（Select one of the following:）	1.Discharged to another hospital 2.Discharged home 3.Death<br>4.Other（ ）

<table>
<tr><th colspan="2">※Palliative Care Phase</th><th colspan="2">※Performance status</th></tr>
<tr><td>1.
Stable</td><td>Patient problems and symptoms are adequately controlled by established plan of care and
•Further interventions to maintain symptom control and quality of life have been planned and
•Family/carer situation is relatively stable and no new issues are apparent</td><td>0</td><td>Fully active, able to carry on all pre-disease performance without restriction.</td></tr>
<tr><td>2.
Unstable</td><td>An urgent change in the plan of care or emergency treatment is required because
•Patient experiences a new problem that was not anticipated in the existing plan of care and/or
•Patient experiences a rapid increase in the severity of a current problem and/or
•Family/carers circumstances change suddenly impacting on patient care</td><td>1</td><td>Restricted in physically strenuous activity but ambulatory and able to carry out work of a light or sedentary nature, e.g., light housework, office work.</td></tr>
<tr><td rowspan="2">3.
Deteriorating</td><td rowspan="2">The care plan is addressing anticipated needs but requires periodic review because
•Patient's overall functional status is declining and
•Patient experiences a gradual worsening of existing problem and/or
•Patient experiences a new but anticipated problem and/or
•Family/carers experience gradual worsening distress that impacts on the patient care</td><td>2</td><td>Ambulatory and capable of all selfcare but unable to carry out any work activities. Up and about more than 50% of waking hours.</td></tr>
<tr><td>3</td><td>Capable of only limited selfcare, confined to bed or chair more than 50% of waking hours.</td></tr>
<tr><td>4. Terminal</td><td>Death is likely within days</td><td>4</td><td>Completely disabled. Cannot carry on any selfcare. Totally confined to bed or chair.</td></tr>
</table>
