# Supplementary figures and images for: Monitoring patients’ symptom improvement in palliative care units using patient-reported outcomes: a multicenter prospective observational study
Source: BMC Palliat Care. 2026 Jan 22;25:44. doi: 10.1186/s12904-026-01990-9 (PMC12910949; doi:10.1186/s12904-026-01990-9)

### Additional file.1

Prevalence and severity of symptoms at admission among all patients

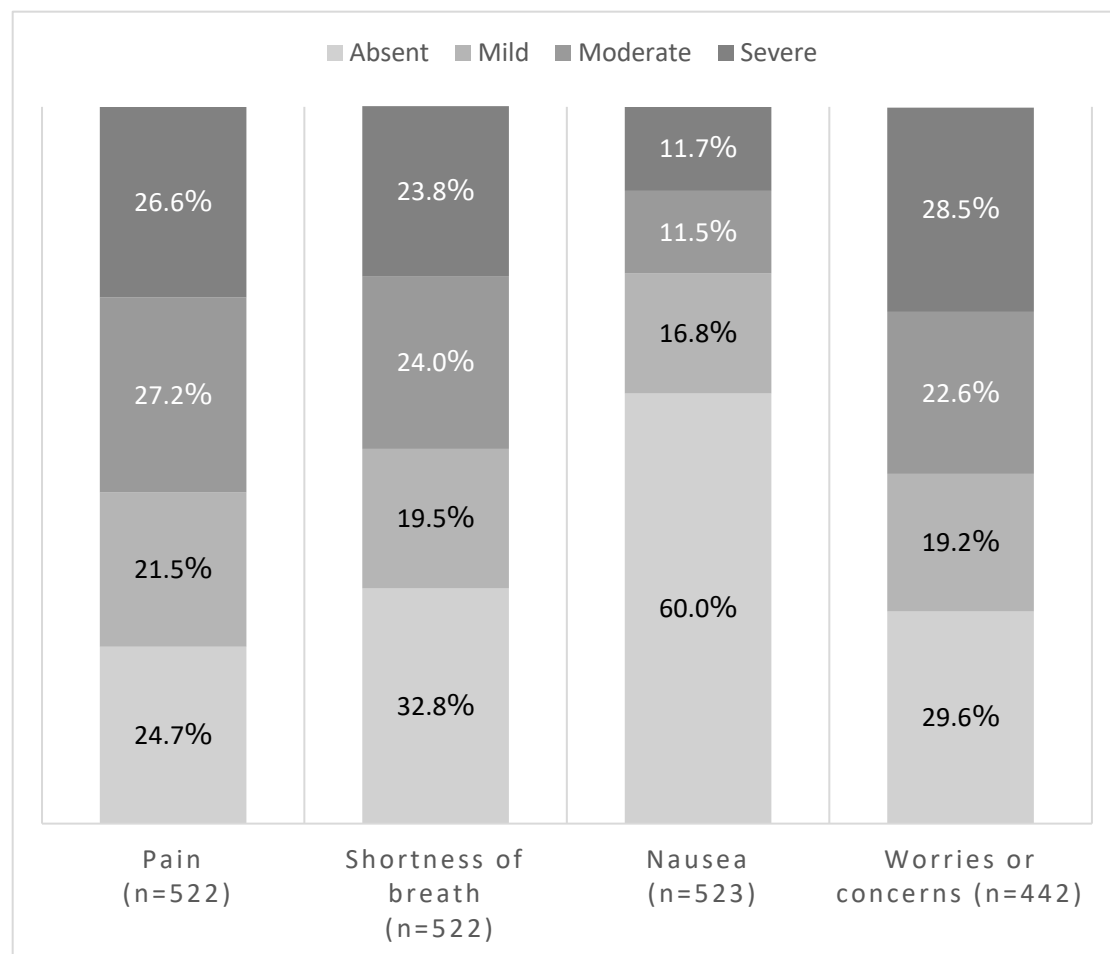

Supplement: Supplementary file 2 — Additional file 1: Prevalence and severity of symptoms at admission among all patients. [file 12904_2026_1990_MOESM2_ESM.pdf]

## Additional file.2

Symptom improvement rates 1 week after Admission among all patients

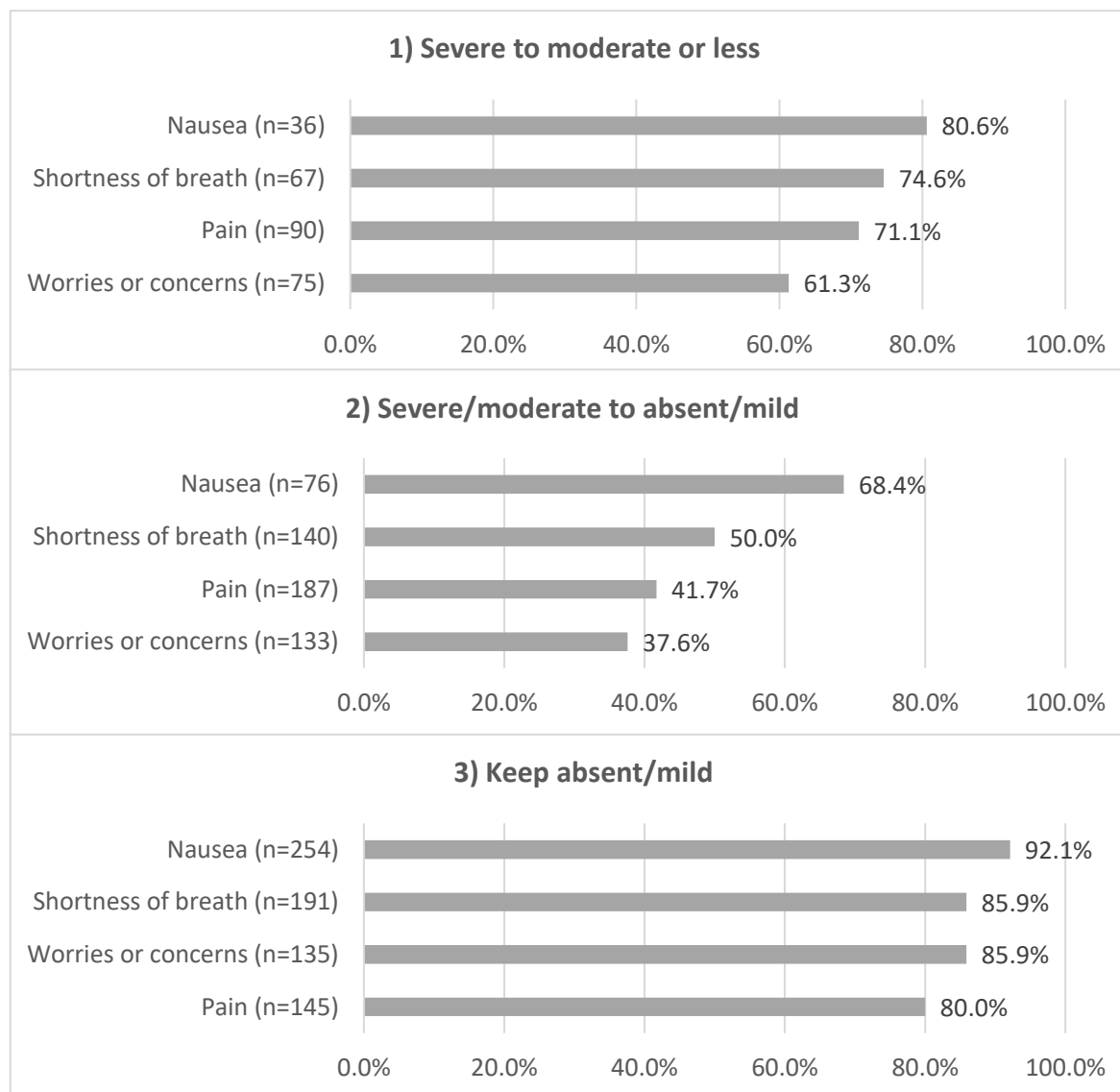

Supplement: Supplementary file 3 — Additional file 2: Symptom improvement rates 1 week after Admission among all patients. [file 12904_2026_1990_MOESM3_ESM.pdf]

### Additional file.3

Changes in IPOS scores from admission to week 4 among all patients

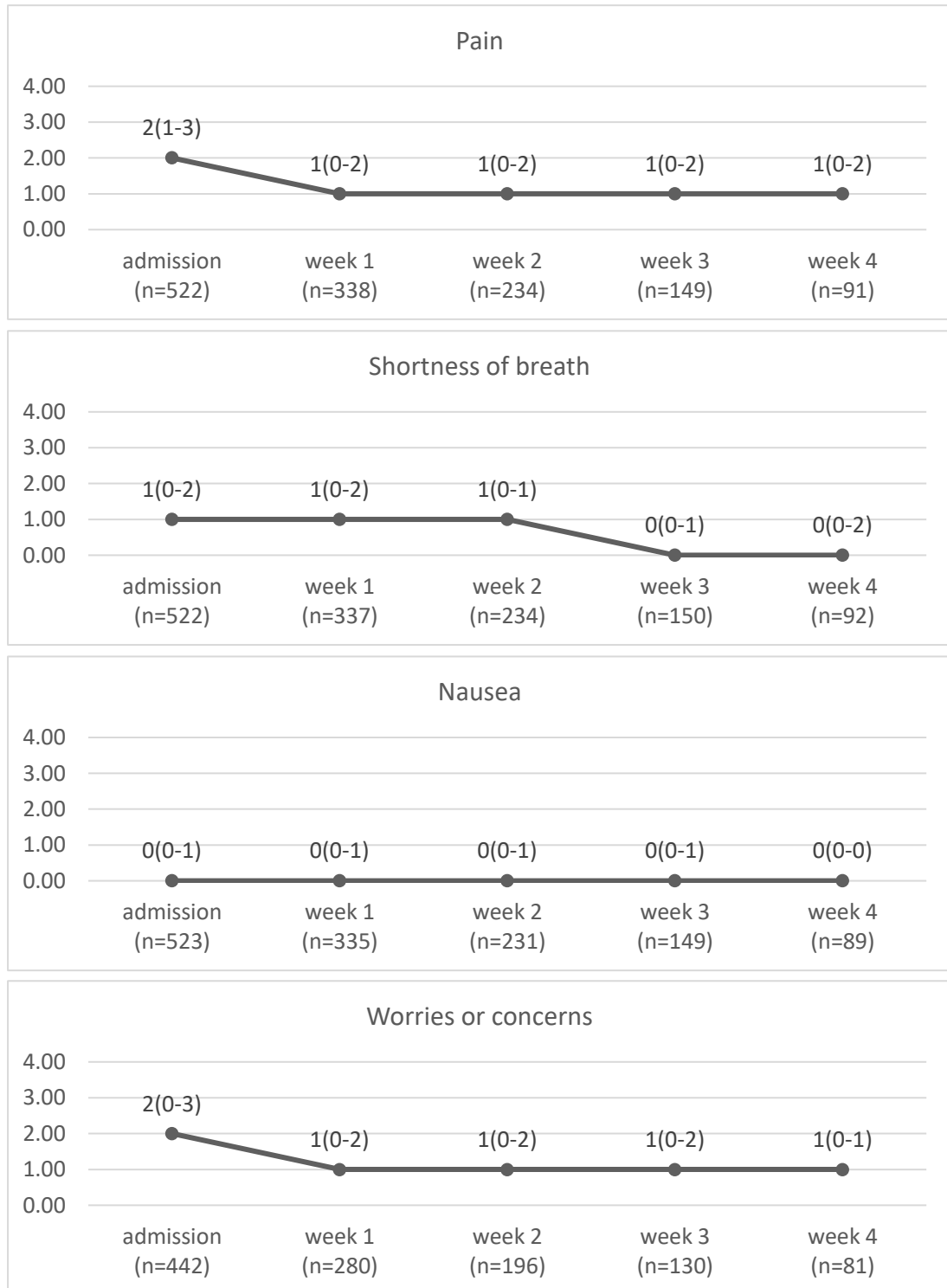

Supplement: Supplementary file 4 — Additional file 3: Changes in IPOS scores from admission to week 4 among all patients. [file 12904_2026_1990_MOESM4_ESM.pdf]
