# Supplementary material for: Monitoring patients’ symptom improvement in palliative care units using patient-reported outcomes: a multicenter prospective observational study
Source: BMC Palliat Care. 2026 Jan 22;25:44. doi: 10.1186/s12904-026-01990-9 (PMC12910949; doi:10.1186/s12904-026-01990-9)
Supplement: Supplementary file 5 — Additional file 4: IPOS scores in patients who completed PRO assessments and died during the following week [file 12904_2026_1990_MOESM5_ESM.pdf]

Additional file. 4

IPOS scores in patients who completed PRO assessments and died during the following week

a. pain

IPOS score	week0		week1		week2		week3	
	n	%	n	%	n	%	n	%
0	19	20.7	13	43.3	8	42.1	3	27.3
1	23	25.0	5	16.7	6	31.6	1	9.1
2	23	25.0	6	20.0	2	10.5	6	54.6
3	14	15.2	2	6.7	2	10.5	1	9.1
4	13	14.1	4	13.3	1	5.3	0	0.0

b. shortness of breath

IPOS score	week0		week1		week2		week3	
	n	%	n	%	n	%	n	%
0	17	18.5	13	43.3	8	42.1	2	18.2
1	16	17.4	8	26.7	2	10.5	2	18.2
2	25	27.2	4	13.3	5	26.3	5	45.5
3	23	25.0	4	13.3	4	21.1	2	18.2
4	11	12.0	1	3.3	0	0.0	0	0.0

c. nausea

IPOS score	week0		week1		week2		week3	
	n	%	n	%	n	%	n	%
0	41	44.6	22	73.3	11	57.9	6	54.6
1	22	23.9	3	10.0	2	10.5	2	18.2
2	12	13.0	3	10.0	2	10.5	2	18.2
3	9	9.8	1	3.3	0	0.0	1	9.1
4	8	8.7	1	3.3	4	21.1	0	0.0

d. worries or concerns

IPOS score	week0		week1		week2		week3	
	n	%	n	%	n	%	n	%
0	22	24.4	14	48.3	3	15.8	1	9.1
1	13	14.4	4	13.8	5	26.3	2	18.2
2	20	22.2	6	20.7	7	36.8	5	45.5
3	20	22.2	1	3.5	2	10.5	2	18.2
4	15	16.7	4	13.8	2	10.5	1	9.1
